# Environmental Burden of Disease due to Emissions of Hard Coal- and Lignite-Fired Power Plants in Germany

**DOI:** 10.3389/ijph.2023.1606083

**Published:** 2023-08-14

**Authors:** Michaela Liebig-Gonglach, Lina Neunhäuserer, Jeroen Kuenen, Barbara Hoffmann, Vanessa Soppa, Volker Diegmann, Claudia Hornberg

**Affiliations:** ^1^ Department of Sustainable Environmental Health Sciences, Medical School East Westphalia-Lippe, Bielefeld University, Bielefeld, Germany; ^2^ IVU Umwelt GmbH, Freiburg, Germany; ^3^ Department of Climate, Air and Sustainability, TNO (Nederlandse Organisatie voor toegepast-natuurwetenschappelijk onderzoek), Utrecht, Netherlands; ^4^ Institute for Occupational, Social and Environmental Medicine, Heinrich Heine University Düsseldorf, Düsseldorf, Germany

**Keywords:** environmental burden of disease, coal-fired power plants, particulate matter (PM_2.5_), nitrogen dioxide (NO_2_), chemical transport model

## Abstract

**Objectives:** The study estimated the environmental burden of disease (EBD) attributable to a long-term exposure of the population to nitrogen dioxide (NO_2_) and fine particulate matter (PM_2.5_) emissions from hard coal- and lignite-fired power plants in Germany for the year 2015.

**Methods:** The contribution of coal-fired power plants to the total air pollutant concentration was modelled using a chemical transport model and then combined with population data to assess the corresponding population exposure. We calculated years of life lost (YLL), years of life with disability, or disability-adjusted life years for different health outcomes with a strong evidence for an association with the exposure.

**Results:** The burden of disease from PM_2.5_ emissions from lignite is 1.2 times higher than that from hard coal emissions (7,866 YLL compared to 6,412 YLL). NO_2_ emissions from lignite, cause a burden of disease 2.3 times higher than hard coal NO_2_-emission (13,537 YLL compared to 5,906 YLL). The EBD for both pollutants is dominated by diseases of the cardiovascular system.

**Conclusion:** Abandoning energy generation by coal-fired power plants would lower the burden of disease in Germany.

## Introduction

Lignite- and hard coal-fired power plants contribute relevant amounts of various pollutants to the environment [1, 2]. In addition to sulfur dioxide (SO_2_), nitrogen oxides (NO_X_) and particulate matter (PM_10_, PM_2.5_), these include heavy metals such as lead, cadmium and mercury. The uptake of these pollutants, by, e.g., breathing polluted air can lead to significant health impairments in humans.

It was shown that air pollution was the fourth leading risk factor for disease and deaths worldwide in 2015, whereby particulate matter (PM_2.5_) in ambient air has the highest share [3, 4]. As indicated by the updated Air Quality Guidelines (AQG) of the World Health Organization (WHO) [5] in 2021, air pollution is still an important risk factor causing adverse health effects down to very low pollutant concentration levels and is responsible for a considerable loss of healthy life years every year [6]. In addition to pollutants from traffic, industry, agriculture and house-firing, coal-fired power plants (CFPP) for energy production are a major source of air pollution in Germany. In recent studies [7–9], the first calculations have been performed at the European level to estimate the burden of disease from CFPP emissions in Europe. However, these reports are limited to the impact of CFPP emissions on natural mortality. There is no assessment of morbidity as in the Global Burden of Disease (GBD) study, which also does not make source-specific assessments. Furthermore, it has not yet been quantified what share of the burden of disease can be attributed to lignite and hard coal combustion in Germany in detail.

The current publication presents the calculated environmental burden of disease (EBD) in Germany attributable to the PM_2.5_ and NO_2_ emissions of hard coal- and lignite-fired power plants, respectively, for the year 2015. The burden of disease was assessed for disease-specific outcomes based on the modelled contributions of hard coal- or lignite-fired power plants to the air pollutant concentration caused by all (known) emission sources, the so-called *total air pollutant concentration*.

## Methods

Two scenarios were considered, investigating the effect of hard coal- and of lignite-fired power plant emissions, respectively, on the EBD. The respective contribution of emissions from coal-fired power plants to the total air pollutant concentration was calculated using the chemical transport model REM-CALGRID (RCG) [10]. The calculated contribution was then spatially combined with the population distribution in Germany to assess the population exposure for the two scenarios. Based on this, the population was categorized into exposure levels which served as input data for calculating the burden of disease.

### Dispersion Modeling and Emission Data

We used the chemical transport model (CTM) RCG [10] to determine the contribution of coal-fired power plants to the total air pollutant concentration. Modeling was carried out with nested modeling domains starting on the European scale and zooming in to the national scale covering Germany and the surrounding countries to account for transboundary effects of European emissions. CTMs include the modeling of chemical processes influencing NO_2_ and ozone levels on various scales as well as the modeling of secondary aerosol formation which plays an important role on the scales considered here.

For the CTM modeling, emission data from coal-fired power plants in Germany was collected from the BUBE-Online system [11]. We used BUBE data from the emission declaration in accordance with the 11th Federal Immission Control Ordinance [12]. As the data is available in a 4-year cycle, we used year 2016 data as the most recent data at the time of the data query. The BUBE data was evaluated with respect to the economic sectors according to NACE (Statistical Classification of Economic Activities in the European Community) to which the coal-fired power-plants are assigned. In this study, the coal-fired power plants attributed to the economic sectors “electricity generation,” “heating and cooling supply” and “treatment and disposal of non-hazardous waste” were considered, comprising the largest part of the total emissions of all coal-fired power plants recorded in the BUBE data. Additionally, the BUBE query yielded relevant data on source-specific variables such as source position and height, flue gas volume flow or flue gas temperature, which were required for the CTM modeling. The emission data of the coal-fired power plants from the BUBE-Online system was scaled to 2015 (Supplementary Table S1), as this was the reference year in the presented study, using annual emission sums from the German Environment Agency (UBA), emissions inventory database. All other emission data in Germany required for CTM modeling was provided by the UBA from the Gridding Emission Tool for ArcGIS [13] in the 2020 submission according to the EEA 2019 [14] for the reference year 2015.

For the European emission data outside of Germany, the European Copernicus Atmosphere Monitoring Service CAMS-REG v2.2.1 inventory [15], a further development of the inventory presented in Kuenen et al. [16] served as the starting point. This inventory contains emissions from all relevant anthropogenic European sources and covers all major air pollutants. The inventory was compiled, as far as possible, using officially reported emissions by source category from each country. In instances where these are inaccurate, incomplete or not reported at all, alternative emission estimates are used. In addition, a consistent spatial distribution was applied. For point sources and in particular power plants, E-PRTR reporting [2] was used in combination with other sources of information on power plants, including Large Combustion Plants (LCP) reporting [17] and information from the commercial database Platts-WEPP [18]. For the purpose of this study, a specific version of CAMS-REG v2.2.1 was made where coal-fired power plants were identifiable as a specific sector.

### Exposure Modeling

Exposure modeling for Germany was carried out based on the spatially distributed contribution of hard coal- or lignite-fired power plants to the total concentration of the pollutants NO_2_ and PM_2.5_ in outdoor air modeled with RCG and on spatially distributed population data. RCG results from the national scale were used at a spatial resolution of 2 × 2 km^2^. Grid cell-based, spatially distributed population data was available for Germany from the 2011 Census [19] with 2011 as the reference year. The population data was aggregated to a 1 × 1 km^2^ grid and scaled to the reference year 2015 for the presented study based on annually available summary population data for Germany from the Federal Health Reporting Information System [20]. The average population size is not subject to significant fluctuations during the reporting period, so no significant difference in the population exposure distribution is expected. Both the spatially distributed contribution of hard coal- or lignite-fired power plants in Germany and the population distribution were spatially combined to assess the population exposure for the two scenarios. Based on this, the population was categorized into different exposure levels providing the number of exposed people (p_i_) in different exposure categories (∆_i_) needed for calculating the burden of disease (Supplementary Table S2).

### EBD Quantification Method

**FIGURE 1:**
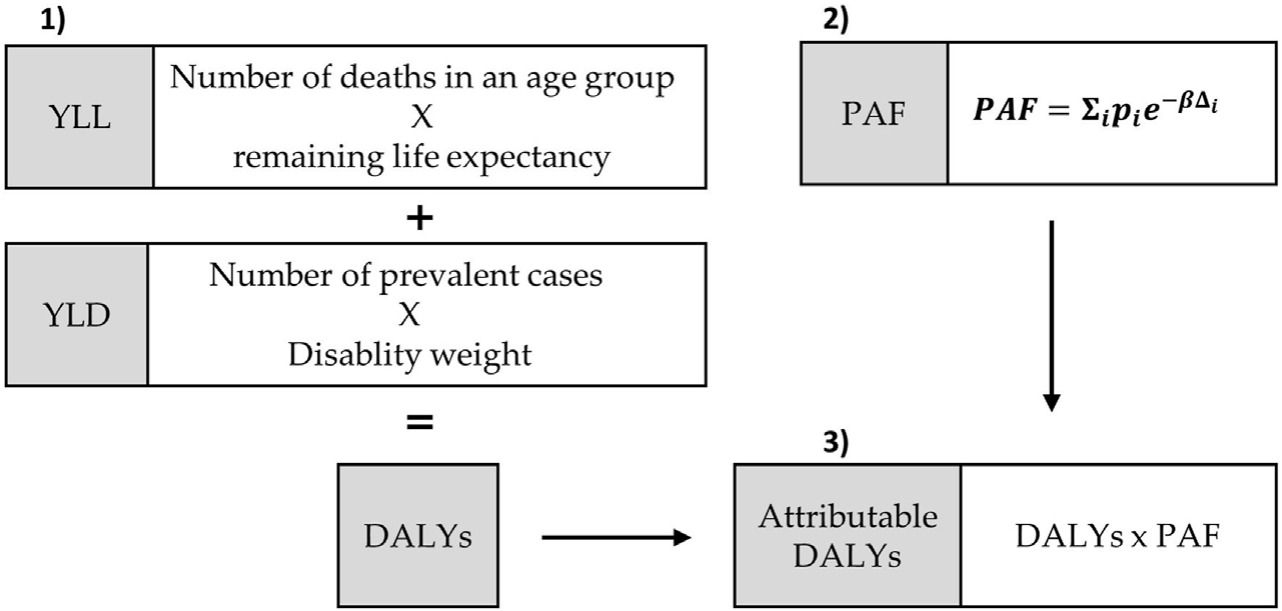
Scheme of steps to quantifiy the environmental burden of disease due to lignite and hard coal-firing in Germany in 2015 ([23], modified). 1) Calculation of YLL (years of life lost due to premature mortality), YLD (years lived with disability) and DALYs (disability-adjusted life years), 2) Calculation of the PAF (population attributable fraction): pi = exposed population, β = linear exposure-response function, Δi = category of exposure, 3) Calculation of attributable DALYs (to the exposure).

The burden of disease caused by the emissions of hard coal- and lignite-fired power plants was assessed by using a standardized framework to quantify population health impacts using the loss of healthy life years [21, 22] (Figure 1). The assessed burden of disease is expressed by the metric Disability-Adjusted Life Years (DALYs) which is calculated as the sum of the mortality component Years of Life Lost due to premature mortality (YLL) and the morbidity component Years Lived with Disability (YLD). YLL are calculated by multiplying the number of deaths in an age group with the average remaining life expectancy in the population (Figure 1). YLD are the product of the number of prevalent cases of a disease and the disability weight. The disability weight is a modeled factor that quantifies the severity of a disease on a scale from 0 (full health) to 1 (a state comparable to death).

To estimate the burden of disease caused by emissions of coal-fired power plants, the population attributable fraction (PAF) has to be determined as described previously [21]. For calculating the PAF, the number of exposed people (p_i_) in different exposure categories (∆_i_) is needed. Additionally, the relative risk for the population to die or to experience a particular disease when exposed to the pollutant, is included into the formula. The calculation of the relative risk assumes a linear relationship between the risk to die or fall ill and a change in the exposure category, which is expressed by a linear exposure-response function (β):

$$PAF=\Sigma_{i}p_{i}e^{-\beta \Delta_{i}}$$

The YLL, YLD or DALYs attributable to the exposure towards NO_2_ or PM_2.5_ were calculated as a product of the YLL, YLD or DALYs caused by the regarded health outcome and the PAF.

All calculations were performed using the R Statistical Environment [23]. The models were run for both sexes separately and stratified by 5-year age groups. The last age group includes all people above the age of 90 years.

### Health Outcomes and Exposure-Response Functions

The burden of disease was calculated for outdoor air pollutants emitted from coal-fired power plants and specific disease outcomes. Exposure-outcome pairs with (1) strong evidence for an association as evaluated by systematic reviews [24–26], (2) high public health relevance, (3) available data for modeling long-term exposure of the German population, and (4) suitable data for disease-specific morbidity and mortality in the German population were selected (Supplementary Tables S3, S4). NO_2_-Exposure-outcome pairs with moderate evidence (diabetes mellitus, stroke, Bronchial asthma) were included in an additional analysis (Supplementary Table S3). For PM_2.5_ we used the outcomes included in the GBD study that chooses exposure-outcome pairs with “convincing” or “probable” evidence for a causal relationship [26]. For NO_2_ we followed a recent burden of disease assessment in Germany for NO_2_ [24] and used the harmonized categories describing the evidence for an association as strong, moderate or weak across the pollutants (Supplementary Tables S3, S4). Where applicable, the evidence ratings were updated, if new evidence of “high certainty for an association” was available [6].

Effect estimates for inclusion in the calculations of disease burden were extracted from recent authoritative reviews used by the WHO AQG 2021, the German Environment Agency, the GBD study, or from the recent large-scale European ELAPSE study (Effects of low-level air pollution), which provides the most recent and relevant information on the exposure-response functions for classical air pollutants in Europe [6, 24–26] (Supplementary Tables S3, S4). If several sources of effect estimates were available, we used those that best reflected German population demographics, mean air pollution exposure levels and baseline morbidity as well as mortality rates. For PM_2.5_, the outcome groups as used in the GBD were applied. These contain the same relative risk estimates for morbidity and mortality in a disease category (coronary heart disease, stroke, chronic obstructive pulmonary disease (COPD), lung cancer, diabetes mellitus) [26]. For NO_2_, separate categories were available for cardiovascular and COPD mortality [6, 25].

For the analysis we used single pollutant effect estimates, as sufficient information from multi-exposure models for all endpoints were not available. Therefore, the PM_2.5_ and NO_2_ results for the same health outcomes cannot be added to prevent double counting.

### Input Data for Calculating Burden of Disease

Health and population data were acquired for the year 2015 (Supplementary Table S5), whereas average life expectancy refers to the period of 2015–2017. Population data and data of life expectancy in Germany were obtained from the Federal Statistical Office [27, 28]. Presented data are stratified by 5-years age groups and sex.

The regarded health outcomes were defined according to the International Statistical Classification of Diseases and Related Health Problems (ICD 10). Mortality data for the included health outcomes diabetes mellitus (DM, ICD 10 Code: E10-14), COPD (ICD 10 Code: J44), cardiovascular (ICD 10 Code I00-I99) and coronary heart disease (CHD, ICD 10 Codes: I20-25), stroke (ICD 10 Codes: I60-69) and lung cancer (ICD 10 Code: C34) were provided by the German Federal Health Monitoring [28]. The case numbers from the cause-of-death statistics are a complete assessment of deaths. The accuracy is high as it follows a standard procedure of coding according to international rules of coding ICD.

In Germany morbidity data such as incidence and prevalence are not officially registered in one database. Thus, the prevalent cases of the included health outcomes were quantified by using prevalence rates estimated in the “Gesundheit in Deutschland aktuell” (GEDA 2014/15) study [29–32]. The prevalence data of certain health endpoints from the representative GEDA survey are not physician diagnoses according to ICD, but subjective statements by affected persons on the 12-month prevalence. Prevalent cases for lung cancer obtained from the Cancer Registry [33] are also a standardized estimate. Because the regarded outcomes are usually not prevalent in children and adolescents, the current study includes only cases of persons aged over 25 years.

The disability weights were calculated using the burden of disease data for Germany in 2015 acquired from the Institute for Health Metrics and Evaluation (IHME) [34]. The IHME provides annual prevalence data and estimated YLD (incl. an uncertainty interval) for many health outcomes and countries. Thus, disability weights for specific health outcomes were assessed by dividing YLD by prevalence.

## Results

### Burden of Disease Attributable to NO_2_ Emissions of Hard Coal- and Lignite-Fired Power Plants

NO_2_ emitted by hard coal-fired power plants caused 5,695 YLL (95% CI 3,893; 7,567) due to cardiovascular mortality and about 211 YLL due to COPD. However, the 95% confidence interval (CI) for COPD ranges from 0 YLL up to 349 YLL (Table 1), thus implying the possibility of a very low number of attributable YLL to the NO_2_ emissions originating from hard coal-fired power plants.

**TABLE 1:** Burden of disease caused by NO_2_ emissions of hard coal- and lignite fired power plants in Germany in 2015.

Health outcome mortality	Hard coal		Lignite	
	Cardiovascular	COPD	Cardiovascular	COPD
PAF % (95% CI)	0.183 (0.125; 0.243)	0.063 (0; 0.105)	0.418 (0.286; 0.555)	0.146 (0; 0.240)
YLL (95% CI)	5,695 (3,893; 7,567)	211 (0; 349)	13.051 (8,935; 17,316)	486 (0; 801)

Health outcomes with strong evidence (cardiovascular mortality and COPD mortality, from the age of 25 years and older).

NO_2_, nitrogen dioxide; PAF, Population attributable fraction; YLL, Years of Life Lost; CI, confidence interval; COPD, chronic obstructive pulmonary disease.

Exposure to NO_2_ emissions from lignite-fired power plants primarily accounts for cardiovascular mortality by 13,051 YLL (95% CI 8,935; 17,316). However, COPD-mortality accounts for 486 YLL, whereas the 95% CI includes the possibility of 0 up to 801 YLL for COPD.

Thus, the largest share of the burden of disease caused by the two selected health outcomes associated with the exposure to NO_2_ emissions of hard coal- and lignite-fired power plants in Germany in 2015, is contributed by cardiovascular mortality that corresponds to 96.3% of all calculated YLL, whereas COPD mortality contributes 3.7%.

Moreover, the results show that the NO_2_ emissions of hard coal-fired power plants cause about 30.4% of the total YLL for cardiovascular and COPD mortality caused by NO_2_ emissions of hard-coal and lignite-fired power plants.

For the health outcomes with only moderate evidence (diabetes mellitus, stroke, and bronchial asthma), a determination of the potential additional burden of disease due to exposure to NO_2_ emissions from coal and lignite-fired power plants in 2015 was made, indicating a relevant additional burden of disease (Table 2). The analysis shows that NO_2_ emissions originating from hard coal-CFPP contribute 254 additional attributable YLL associated with diabetes, with a confidence interval of 0 YLL up to 713 YLL (Table 2). With 582 YLL, more than twice as many attributable YLL associated with diabetes were estimated for NO_2_ emissions of lignite-fired power plants, again with a wide confidence interval (95% CI) including the possibility of 0 YLL to 1,617 YLL.

**TABLE 2:** Burden of disease attributable to NO_2_ emissions of hard coal- and lignite-fired power plants in Germany in 2015.

Health outcome mortality	Hard coal	Lignite				
	Diabetes mellitus	Diabetes mellitus		
PAF % (95% CI)	0.235 (0; 0.660)	0.538 (0; 1.495)				
YLL (95% CI)	254 (0; 713)	582 (0; 1,617)				

Health outcome morbidity	Hard coal			Lignite		
	Diabetes mellitus	Stroke	Bronchial asthma	Diabetes mellitus	Stroke	Bronchial asthma
PAF % (95% CI)	0.295 (0.051; 0.541)	0.006 (0; 0.090)	0.485 (0.002; 0.965)	0.675 (0.117; 1.229	0.015 (0; 0.207)	1.105 (0.005; 2.172)
YLD (95% CI)	1,101 (189; 2,015)	11 (0; 150)	580 (3; 1,153)	2,516 (435; 4,580)	25 (0; 345)	1,320 (6; 2,595)

Health outcomes with moderate evidence for an association for mortality (diabetes mellitus) and morbidity (diabetes mellitus, stroke, bronchial asthma), from the age of 25 years and older.

NO_2_, nitrogen dioxide; PAF, Population attributable fraction; YLL, Years of Life Lost; YLD, years lived with disability; F; CI, confidence interval; COPD, chronic obstructive pulmonary disease.

Considering the potential additional YLD due to NO_2_-emissions from hard coal-fired power plants, diabetes mellitus, stroke, and bronchial asthma contribute a total of 1,692 YLD to the burden of disease in the age groups 25 years and older (Table 2). For NO_2_-emissions of lignite-fired power plants, the total additional contribution is 3,861 YLD.

### Burden of Disease Attributable to PM_2.5_ Emissions of Hard Coal- and Lignite-Fired Power Plants

Concerning the selected health outcomes, the total numbers of calculated YLL resulting from PM_2.5_ emissions of either hard coal- or lignite-fired power plants, are 6,412 YLL from hard-coal (Table 3) and 7,866 YLL from lignite (Table 4). In detail, PM_2.5_ from hard coal accounts for 3,190 YLL (95% CI 2,177; 4,206) due to CHD and 1,888 YLL (95% CI 1,356; 2,456) due to lung cancer (Table 3), whereas lignite CFPP-emissions cause 3,913 YLL (95% CI 2,671; 5,159) due to CHD and 2,317 YLL (95% CI 1,664; 3,013) due to lung cancer (Table 4).

**TABLE 3:** Burden of disease caused by particulate matter PM_2.5_ emissions of hard coal-fired power plants in Germany in 2015.

Health outcome	Coronary heart disease	COPD	Diabetes mellitus	Stroke	Lung cancer	Sum
PAF % (95% CI)	0.250 (0; 0.329)	0.153 (0; 0.194)	0.19 (0; 0.217)	0.265 (0; 0.319)	0.161 (0; 0.209)	
YLL (95% CI)	3,190 (2,177; 4,206)	572 (414; 725)	210 (155; 235)	552 (412; 663)	1,888 (1,356; 2,456)	6,412
YLD (95% CI)	232 (159; 306)	385 (278; 487)	727 (537; 814)	472 (352; 567)	9 (6; 12)	1,825
DALYs (95% CI)	3,422 (2,335; 4,513)	956 (692; 1,212)	937 (691; 1,048)	1,024 (764; 1,230)	1,897 (1,363; 2,467)	8,236

Health outcomes with strong evidence for an association (diabetes mellitus, COPD, coronary heart diseases, stroke and lung cancer) from the age of 25 years and older.

PM_2.5_, particulate matter; PAF, Population attributable fraction; YLL, Years of Life Lost; YLD, Years Lived with Disability; DALYs, Disability Adjusted Life Years; CI, confidence interval.

**TABLE 4:** Burden of disease attributable to PM_2.5_ emissions of lignite-fired power plants in Germany in 2015.

Health outcome	Coronary heart disease	COPD	Diabetes mellitus	Stroke	Lung cancer	Sum
PAF % (95% CI)	0.306 (0.209; 0.404)	0.188 (0.136; 0.239)	0.24 (0.175; 0.266)	0.326 (0.243; 0.391)	0.197 (0.142; 0.256)	
YLL (95% CI)	3,913 (2,671; 5,159)	702 (507; 889)	257 (190; 288)	677 (505; 813)	2,317 (1,664; 3,013)	7,866
YLD (95% CI)	285 (195; 376)	472 (341; 598)	892 (658; 998)	579 (432; 696)	11 (8; 14)	2,239
DALYs (95% CI)	4,199 (2,865; 5,535)	1,173 (849; 1,487)	1,149 (848; 1,286)	1,256 (937; 1,509)	2,328 (1,672; 3,027)	10,105

Health outcomes with strong evidence (diabetes mellitus, COPD, coronary heart diseases, stroke and lung cancer) from the age of 25 years and older.

PM_2.5_, particulate matter; PAF,Population attributable fraction; YLL, Years of Life Lost; YLD, Years Lived with Disability; DALYs, Disability Adjusted Life Years; CI, confidence interval; COPD, chronic obstructive pulmonary disease.

Regarding the percentages of YLL attributable to PM_2.5_ emissions of hard coal- or lignite-fired power plants, for both types of coal it is dominated by YLL due to CHD which accounts for about 50% of the YLL and lung cancer contributing about 30% to the overall YLL caused by hard-coal or lignite, respectively (Tables 3, 4).

Beyond that, 8.9% of the total assessed YLL were found to be a consequence of COPD and 8.6% of stroke, whereas diabetes mellitus contributes less than 3.3% to the total YLL caused by PM_2.5_ emissions of hard coal- or lignite-fired power plants, respectively (Tables 3, 4).

In terms of the estimated years lived with disability (YLD) 1,825 YLD were attributable to PM_2.5_ emitted by hard coal- and 2,239 YLD by lignite-fired power plants. For both types of coal YLD predominantly result from diabetes mellitus which accounts for 39.8% of the total assessed YLD. The corresponding value related to hard coal is 727 YLD (95% CI 537; 814) (Table 3) and for lignite 892 YLD (95% CI 658; 998) (Table 4). Beyond that, preventable years lived with disability resulting from stroke constitute 25.9% of the YLD, COPD constitutes 21.1% and CHD 12.7% of the YLD, respectively.

The calculated burden of disease metric DALYs yields 8,236 attributable to PM_2.5_ emissions of hard coal (Table 3)–and 10,105 DALYs attributable to PM_2.5_ emissions of lignite-fired power plants (Table 4). As shown by the DALYs for the various health outcomes considered here, PM_2.5_ emissions from hard coal and lignite-fired power plants contributed primarily to the disease burden of CHD in 2015, accounting for about 42% of the DALYs estimated for PM_2.5_. This corresponds to 3,190 YLL by hard coal and 3,913 by lignite PM_2.5_ emissions. The burden of disease due to lung cancer accounts for 23% of the DALYs, whereas diabetes mellitus, COPD and stroke account for 11%–12% of the DALYs. In contrast to the other health outcomes considered here, DALYs due to diabetes mellitus predominantly result from morbidity, i.e., the assessed YLD due to diabetes mellitus contribute 78% to the DALYs.

## Discussion

### Burden of Disease due to CFPP

To the knowledge of the authors, this is the first study estimating the burden of disease differentiated for PM_2.5_ and NO_2_ emissions from hard coal- and lignite-fired power plants in Germany. For the calculation of the burden of disease due to emissions from CFPP in Germany into outdoor air, the disease-specific approach was chosen [24, 26]. In this approach, burden of disease is calculated for selected diseases for which strong evidence for an association with the respective exposure is available. Moreover, this approach considers the effects on different health outcomes and the contribution of mortality and morbidity metrics to the total burden of disease.

The results show that CFFP in Germany contributed to a substantial burden of disease from cardiovascular disease, COPD, diabetes mellitus, stroke and lung cancer in 2015 in the German population.

When comparing the DALYs for the selected health outcomes caused by PM_2.5_ emissions of CFFPs in the year 2015, about 19% fewer DALYs for hard coal than for lignite were estimated.

With regard to the effects of long-term NO_2_-exposure on the selected cardiovascular or COPD mortality in the population, respectively, the current results show that the emissions of lignite-fired power plants cause 2.3 as much YLL than the emissions of hard-coal-fired power plants while the difference for PM_2.5_ is considerably smaller at 1.2.

Moreover, the results for PM_2.5_ and NO_2_ that refer to the selected endpoints with strong evidence show that the main burden of disease arise from diseases of the cardiovascular system.

Overall, it can be concluded that in 2015, with respect to the considered health endpoints associated with PM_2.5_ and NO_2_, lignite-fired power plants caused a greater disease burden than emissions from hard coal-fired power plants. This difference can be attributed to the approximately 2-fold higher annual emission of the pollutants NO_2_ and PM_2.5_ from lignite-compared to hard coal-fired power plants (Supplementary Table S1). Thus, in Germany especially the shut-down of lignite fired power plants in particular may avoid a substantial burden of disease on a population level with regard to the pollutants PM_2.5_ and NO_2_.

### Comparability to Other Studies

For the disease burden from hard coal- and lignite-fired power plants determined in this study, there are little comparable burden of disease estimations available from previous studies that would allow a direct comparison. The results of a study that reported attributable deaths from CFPP emissions in Europe in 2013 [8] are not comparable because that study identified only natural mortality effects, which were not included in our study.

In the present study, the burden of disease was exclusively calculated for the additional PM_2.5_ and NO_2_ air pollution exposure caused by hard coal- or lignite-fired power plant emissions. The total burden of disease in the population due to the exposure to PM_2.5_ or NO_2_ total air pollution concentration originating from all emission sources, including industry, agriculture, traffic, or domestic heating was not included. This source-specific approach sets this study apart from the GBD study [26] or a prior publication that assessed the burden of disease due to the exposure to NO_2_ background air pollution in Germany in 2015 [24]. A calculation of the relative fraction of disease burden due to NO_2_ emissions from CFPP compared to the total burden of disease due to NO_2_ background air pollution in the cited study [24] is not possible, because the present study used updated information on the strength of the evidence for exposure-response analyses and for effect estimates. These updates led to the inclusion of COPD mortality, for which evidence was considered as strong in a recent systematic review [25] and to a higher effect estimate for cardiovascular mortality from ELAPSE [6] than in the prior publication [24].

### Single Pollutant Effects

The pollutants PM_2.5_ and NO_2_ are typically positively correlated, which may lead to an overestimation of the disease burden, if the calculated metrics for both pollutants were simply added up using single-exposure estimates from health effects studies. When estimating the burden of disease caused by CFPP, it is important to consider that the attributable shares of the burden of disease caused by PM_2.5_ and NO_2_ may only be added up when using estimates from multi-exposure models. In the current analysis of disease-specific endpoints, effect estimators from single-pollutant models were used that did not control for the co-occurrence of concomitant pollutants such as NO_2_ or PM_2.5_ [26]. That is, the effect estimators used for PM_2.5_ are not adjusted for NO_2_ and *vice versa*. An addition of the disease burdens that were determined in the analysis presented here would therefore lead to an overestimation of the disease burden that is difficult to quantify, since the respective contribution of the individual pollutants to the disease burden cannot be differentiated.

### Study Limitations

For various reasons, the attributable burden of disease associated with emissions from CFPP in Germany, which were investigated in this study, represents only a part of the total burden of disease originating from this source.

Thus, the attributable burden of disease within this project represents an underestimation of true effects, because emitted pollutants that are not primarily airborne, but may also account for a substantial health impact, were not included, due to a lack of sufficient exposure data for the German population. Heavy metals form an important group of health relevant pollutants that predominantly enter humans through ingestion of contaminated foods or dermal absorption. A selective population-level exposure to heavy metals emitted from coal-fired-power plants cannot be reliably estimated due to the complex transmission pathways of these pollutants.

Moreover, warm season ozone, a secondary pollutant formed from emitted precursor substances under conditions of high ultraviolet radiation, and associated COPD mortality was not included because of a lack of season-specific mortality rates.

In the presented analysis, health impacts for which there is strong evidence of an exposure-effect relation were included. Whether outcomes with a moderate evidence as shown for NO_2_ contribute to a critical extent is not certain.

The validity of EBD-results strongly depends on the quality, availability and comparability of the input-data. Because of the prevalence estimates used of certain health outcomes captured by subjective statements of 12-month prevalence by those affected, some uncertainties must also be assumed in the results of burden of disease or morbidity metrics. Moreover, in the current study, relevant health outcomes had to be excluded due to a lack of representative health data.

Moreover, the cross-border transport of emissions from Germany into neighboring European countries was not considered in this study, therefore, the burden of disease in neighboring countries associated with the emissions originating from coal-fired power plants in Germany was not assessed. However, there is some evidence from a Europe-wide study [8] that transboundary exchange of pollution contributes substantially to the burden of disease in neighboring countries, in general. Therefore, it can be assumed that modeling a shutdown of all coal-fired power plants in Germany in 2015 would also have yielded significant health gains in neighboring countries.

### Conclusion

Abandoning energy generation by coal-fired power plants would reduce hazardous pollution and lower the burden of disease in Germany and beyond, including its neighboring European countries. In particular, the shutdown of lignite-fired power plants would contribute to a major reduction of the burden of disease caused by emissions from coal-fired power plants in Germany. But, even hard coal-fired power plants contribute substantially to the burden of disease in the population of Germany.

Consequently, the coal phase-out that has already been politically decided for Germany should not be delayed, not only to reduce the direct emission-related burden of disease, but also to make a decisive contribution to global climate protection at the same time.
