# Supplementary material for: Environmental Burden of Disease due to Emissions of Hard Coal- and Lignite-Fired Power Plants in Germany
Source: Int J Public Health. 2023 Aug 14;68:1606083. doi: 10.3389/ijph.2023.1606083 (PMC10460906; doi:10.3389/ijph.2023.1606083)
Supplement: Supplementary file 1 [file DataSheet1.pdf]

International Journal of Public Health

Environmental burden of disease due to emissions of hard coal- and lignite-fired power plants in Germany

Supplement

Table S1 NO_x- and $PM_{2.5}$-emissions of coal-fired power plants from BUBE-Online (1) scaled to the reference year 2015, separated by hard coal-fired power plants, lignite-fired power plants and power plants burning hard coal and lignite.

Substance	Hard coal [kg]	Lignite [kg]	Hard coal and Lignite [kg]	Sum# [kg]
NO_X	57,056,034	114,608,66	4,431,521	176,096,121
PM2.5	711,153	1,646,663	58,663	2,416,479

the non-rounded values were used for the summation

Table S2 Number of exposed inhabitants (p_i) in Germany by NO_2 and $PM_{2.5}$-emissions from lignite and hard coal-fired power plants in different concentration classes (Δ_i). Modelled distribution in the total population for the year 2015.

	NO_2		$PM_{2.5}$	
Concentration classes [µg/m³]	Hard coal	Lignite	Hard coal	Lignite
	[number of exposed inhabitants (p_i)]			
0 <= x < 0.1	26,969,413	24,125,840	29,490,596	29,188,576
0.1 <= x < 0.2	30,279,877	14,017,266	51,973,316	31,687,830
0.2 <= x < 0.3	16,105,511	7,900,029	221,408	14,632,914
0.3 <= x < 0.4	5,210,562	9,642,426	1,313	3,865,990
0.4 <= x < 0.5	1,758,738	5,489,506	0	1,696,337
0.5 <= x < 0.6	822,219	2,876,480	0	372,405
0.6 <= x < 0.7	218,243	2,499,365	0	142,079
0.7 <= x < 0.8	118,159	2,314,450	0	47,970
0.8 <= x < 0.9	85,074	1,723,703	0	17,564
0.9 <= x < 1	17,035	1,836,087	0	9,829
1 <= x < 1.2	79,271	2,619,193	0	16,321
1.2 <= x < 1.4	11,356	1,604,510	0	3,865
1.4 <= x < 1.6	8,349	1,165,379	0	226
1.6 <= x < 1.8	298	990,071	0	4,300
1.8 <= x < 2	1,262	588,810	0	110
2 <= x < 2.5	1,266	1,061,314	0	313
2.5 <= x < 3	0	615,121	0	0
3 <= x < 3.5	0	260,854	0	4
3.5 <= x < 4	0	149,144	0	0
4 <= x < 4.5	0	78,171	0	0
4.5 <= x < 5	0	22,160	0	0
5 <= x < 5.5	0	46,144	0	0
5.5 <= x < 6	0	21,299	0	0
6 <= x < 6.5	0	9,513	0	0
6.5 <= x < 7	0	5,402	0	0

	NO_2		$PM_{2.5}$	
Concentration classes [µg/m³]	Hard coal	Lignite	Hard coal	Lignite
	[number of exposed inhabitants (p_i)]			
7 <= x < 7.5	0	717	0	0
7.5 <= x < 8	0	2,447	0	0
8 <= x < 8.5	0	4,110	0	0
8.5 <= x < 9	0	8,492	0	0
9 <= x < 9.5	0	40	0	0
9.5 <= x < 10	0	0	0	0
10 <= x < 11	0	4,721	0	0
11 <= x < 12	0	23	0	0
12 <= x < 13	0	3,842	0	0
13 <= x < 14	0	0	0	0
14 <= x < 15	0	4	0	0
15 <= x < 16	0	0	0	0
16 <= x < 17	0	0	0	0
17 <= x < 18	0	0	0	0
18 <= x < 19	0	0	0	0

Table S3 Effect estimates for health outcomes (mortality and morbidity) with strong and moderate evidence for a causal relation with an exposure to NO_2 [per 10 µg/m³ NO_2].

Health outcome	Outcome type	Evidence for an association	Effect estimates (95 % CI)	Source for effect estimate
Cardiovascular	mortality	strong	1.089 (1.060-1.120)	(2)
COPD	mortality	strong	1.03 (1.00-1.05)	(3)
Diabetes mellitus	mortality	moderate	1.116 (0.915-1.362)	(4)
Diabetes mellitus	morbidity	moderate	1.148 (1.024-1.288)	(4)
Stroke	morbidity	moderate	1.003 (0.964-1.043)	(4)
Bronchial asthma	morbidity	moderate	1.255 (1.001-1.573)	(4)

COPD: Chronic obstructive pulmonary disease; CI: confidence interval

Table S4 Effect estimates for health outcomes with strong evidence (mortality and morbidity) for a causal relation with an exposure to $PM_{2.5}$ [per 1 μg/m³].

Health outcome	Outcome type	Evidence for an association	Effect estimates (95 % CI)	Source for effect estimate
Coronary heart disease	mortality and morbidity	strong	1.015 (1.010, 1.020)	(5)
Stroke	mortality and morbidity	strong	1.016 (1.012, 1.020)	(5)
COPD	mortality and morbidity	strong	1.009 (1.007, 1.012)	(5)
Lung cancer	mortality and morbidity	strong	1.010 (1.007, 1.013)	(5)
Diabetes mellitus	mortality and morbidity	strong	1.012 (1.009, 1.013)	(5)

COPD: Chronic obstructive pulmonary disease; CI: confidence interval

Table S5 Input data used for calculating the environmental burden of disease caused by $PM_{2.5}$ and NO_2 emissions of hard coal- and lignite-fired power plants

Health outcome	ICD 10 Code	Mortality	Morbidity GEDA 2014/15	Disability weight (UI lower; upper values)[1]
Cardiovascular and coronary heart disease	I00-I99 and I20-25)	Cause of death register 2015	(6)	0.027 (0.019; 0.035)
COPD	J44	Cause of death register 2015	(7)	0.064 (0.054; 0.072)
Diabetes mellitus	E11	Cause of death register 2015	(8)	0.070 (0.052; 0.089)
Stroke	I60-69	Cause of death register 2015	(9)	0.155 (0.120; 0.181)
Lung cancer	C34	Cause of death register 2015	(10)	0.152 (0.119; 0.181)

ICD 10: International Statistical Classification of Diseases and Related Health Problems; COPD: Chronic obstructive pulmonary disease; UI: uncertainty interval; [1] (11);

References

1. Betriebliche Umweltdatenberichterstattung (BUBE). BUBE Online (Betriebliche Umweltdaten Bericht-Erstattung) im PRTR. https://www.bube-online.org/.

2. Brunekreef B, Strak M, Chen J, Andersen ZJ, Atkinson R, Bauwelinck M et al. Mortality andMorbidity Effects of Long-Term Exposure to Low-Level PM2.5, BC, NO2, and O3: An Analysis ofEuropean Cohorts in the ELAPSE Project. Boston (2021). Research Report [cited 2021 Sep 20].

3. Huangfu P, Atkinson R. Long-term exposure to NO2 and O3 and all-cause and respiratory mortality: A systematic review and meta-analysis. Environ (Int 2020) 144:105998.

4. Schneider A, Cyrys J, Breitner S, Kraus U, Peters A, Diegmann V et al. Quantifizierung von umweltbedingten Krankheitslasten aufgrund der Stickstoffoxid-Exposition in Deutschland (2018).

5. Murray CJL, Aravkin AY, Zheng P, Abbafati C, Abbas KM, Abbasi-Kangevari M et al. Global burden of 87 risk factors in 204 countries and territories, 1990–2019: a systematic analysis for the Global Burden of Disease Study 2019. The Lancet (2020) 396(10258):1223–49.

6. Busch MA, Kuhnert R. 12-Monats-Prävalenz einer koronaren Herzkrankheit in Deutschland. Journal of Health Monitoring (2017) 2(1):64–9.

7. Steppuhn H, Kuhnert R, Scheidt-Nave C. 12-Monats-Prävalenz der bekannten chronisch obstruktiven Lungenerkrankung (COPD) in Deutschland. Journal of Health Monitoring (2017) 3(2):46–54.

8. Heidemann C, Kuhnert R, Born S, Scheidt-Nave C. 12-Monats-Prävalenz des bekannten Diabetes mellitus in Deutschland. Journal of Health Monitoring (2017) 2(1):48–56.

9. Busch MA, Kuhnert R. 12-Monats-Prävalenz von Schlaganfall oder chronischen Beschwerden infolge eines Schlaganfalls in Deutschland. Journal of Health Monitoring (2017) 2(1):70–6.

10. ZfKD. Krebs - Datenbankabfrage: Zentrum für Krebsregisterdaten (2019). https://www.krebsdaten.de/Krebs/SiteGlobals/Forms/Datenbankabfrage/datenbankabfrage_stufe2_form.html.

11. IHME. VizHub - GBD Results (2022) URL: https://vizhub.healthdata.org/gbd-results/. [Assessed May 19, 2022].
